# Complete chloroplast genome sequence of *Sphaerophysa salsula* (Leguminosae)

**DOI:** 10.1080/23802359.2020.1869618

**Published:** 2021-02-08

**Authors:** Wen-Rui Qu, Pei-Pei Jiao, Xi Jiang, Shan-He Zhang, Tian-Ge Yang, Zhi-Jun Li, Zhi-Hua Wu

**Affiliations:** a Key Laboratory of Protection and Utilization of Biological Resources in Tarim Basin Xinjiang Production and Construction Corps, Tarim University, Alar, China; b College of Life Science, Tarim University, Alar, China; c College of Life Science and Technology of Huazhong Agricultural University, Wuhan, China; d College of Plant Science, Tarim University, Alar, China; e College of Life Sciences, Hubei Provincial Key Laboratory for Protection and Application of Special Plant Germplasm in Wuling Area of China, South-Central University for Nationalities, Wuhan, China

**Keywords:** *Sphaerophysa salsula*, Leguminosae, chloroplast genome, evolution

## Abstract

*Sphaerophysa salsula* (Pall.) DC. is a perennial herbaceous plant belonging to the genus *Sphaerophysa*, Galegeae, Leguminosae, and is mainly distributed in dry areas in Central Asia and Northwest China. The complete chloroplast genome with a total size of 123,300 bp was reported in this study. Further annotation revealed the chloroplast genome contains 109 genes, including 76 protein coding genes, 29 tRNA genes, and four rRNA genes. A total of 107 simple sequence repeats (SSRs) from mononucleotide to hexa-nucleotide repeat motif were identified in the chloroplast genome. This information will be useful for study on the evolution and genetic diversity of *Sphaerophysa salsula* in the future.

*Sphaerophysa salsula* (Pall.) DC., is a highly nutritive and drought-tolerant perennial grass belonging to the genus *Sphaerophysa* (family Leguminosae), and mainly distributed in Central Asia and Northwest China. There are only two species in this genus worldwide, and only one species was found in China. Metabolites extracted from *S. salsula* whole herbs or seeds have many pharmacological activities including bactericidal (Ma et al. 2002), insecticidal (Li et al. 2012), antioxidative (Venkateswarlu et al. 2003), and anti-tumor effects (Wang and Ma 2009). In addition, *S. salsula* usually grows in dry and saline soils in the desert regions or salt pond in Northwest China, it also grows as forage in winter in hillsides and grasslands. Thus, it is an ecologically and economically important plant.

Most chloroplast genomes are characterized by a quadripartite structure, with two copies of an inverted repeat (IR) separating the large (LSC) and small (SSC) single copy regions. A number of previous studies have examined the phylogenetic distribution of different plastid genome rearrangements among legumes, including the loss of one copy of the IR (Palmer and Thompson 1982; Lavin et al. 1990; Jansen et al. 2008). The probably most dramatic example of the phylogenetic utility of a plastid genomic rearrangement among legumes is the loss of one copy of the IR by all members sampled from the tribes Carmichaelieae, Cicereae, Hedysareae, Trifolieae, Fabeae, Galegeae, and three genera of Millettieae (Lavin et al. 1990; Liston 1995; Jansen et al. 2008). The monophyly of this clade with the loss of one copy of the IR is known as the IR-lacking clade or IRLC (Wojciechowski et al. 2000). In this study, to obtain the new insight into the phylogeny of *S. salsula*, we assembled and annotated the accurate chloroplast genome from sequenced data of *S. salsula* with Illumina HiSeq platform.

The materials of *S. salsula* in this study were collected from Atushi City, Kizilsu Kirghiz Autonomous Prefecture, Xinjiang province of China (76°58.491′E, 40°63.411′N, 1662 m above sea level). The voucher specimen (TD-00637, *Sphaerophysa salsula* (Pall.) DC.) was stored in the herbarium of Tarim University. First, the total genomic DNA was extracted using CTAB method and sequenced using the Illumina HiSeq platform. Then, we removed the adaptors and reads with low quality from the raw data (SRR12875154), and the whole chloroplast genome was assembled using GetOrganelle (Jin et al. 2020). Finally, the chloroplast gene structures were annotated using CPGAVAS2 (Shi et al. 2019) and PGA (Qu et al. 2019). The complete chloroplast genome was 123,300 bp (MW122834), the average GC content was 34.09%. The chloroplast genomes encoded 109 functional genes, including 76 protein-coding genes (Supplementary Table S1), 29 tRNA genes, and four rRNA genes. A total of 107 simple sequence repeat (SSR) markers ranging from mononucleotide to hexa-nucleotide repeat motif were identified in *S. salsula* chloroplast genome.

In our study, to explore the phylogenetic relationship of *S. salsula* within Leguminosae, additional 33 species from Leguminosae were studied. With the *Polygala japonica* and *Polygala tenuifolia* as the outgroups, the phylogenetic trees were built from the whole protein-coding gene matrix by maximum-likelihood (ML) and Bayesian inference (BI) (Figure 1). The ML tree was generated using IQ-TREE (Nguyen et al. 2015) based on the best model of TVM + F+R3 and 1000 bootstrap replicates, and BI analysis was performed in MrBayes-3.2.7. This result showed that the analyzed *S. salsula* was closer to the species of *Lessertia frutescens*, both of which belong to the tribe Galegeae. The chloroplast genome of *S. salsula* owns the same gene number (109) as that of *Medicago truncatula* (the model legume) with just one different gene, *ycf68* in *M*. *truncatula* instead of *ycf4* in *S. salsula*. In the phylogenetic tree (Figure 1), the tribe Galegeae with *S. salsula* showed closely related to the tribe Hedysareae, but was distant from the tribe Trifolieae including *M*. *truncatula*.

**Figure 1.**
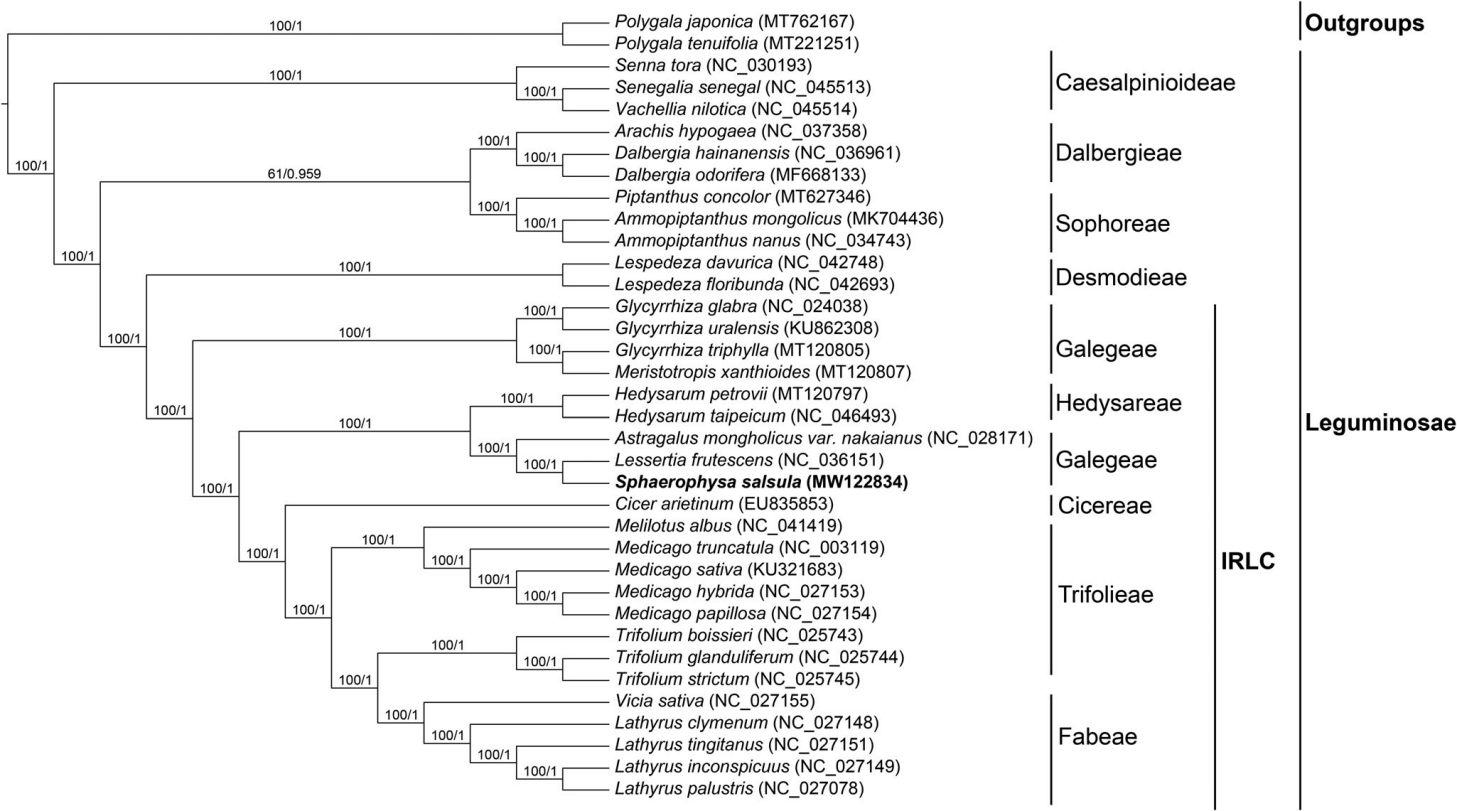
Phylogenetic tree reconstructed by maximum-likelihood (ML) and Bayesian inference (BI) analysis based on the whole chloroplast protein-coding genes of these 36 species.

## Data Availability

The genome sequence data that support the findings of this study are openly available in GenBank of NCBI at https://www.ncbi.nlm.nih.gov/ under the accession no. MW122834. The associated ‘BioProject’, ‘SRA’, and ‘Bio-Sample’ numbers are PRJNA670257, SRR12875154, and SAMN16491006, respectively.
